# Comparison of Teenagers' Early Same-Sex and Heterosexual Behavior: UK Data From the SHARE and RIPPLE Studies

**DOI:** 10.1016/j.jadohealth.2010.05.010

**Published:** 2011-01

**Authors:** Alison Parkes, Vicki Strange, Daniel Wight, Chris Bonell, Andrew Copas, Marion Henderson, Katie Buston, Judith Stephenson, Anne Johnson, Elizabeth Allen, Graham Hart

**Affiliations:** a MRC, Social and Public Health Sciences Unit, University of Glasgow, Glasgow, United Kingdom; b Social Science Research Unit, Institute of Education, University of London, London, United Kingdom; c Department of Social and Environmental Health Research, School of Hygiene and Tropical Medicine, London, United Kingdom; d Department of Infection and Population Health, UCL Centre for Sexual Health and HIV Research, University College London, London, United Kingdom; e Hub for Trials Methodology Research, MRC Clinical Trials Unit, London, United Kingdom; f Medical Statistics Unit, School of Hygiene and Tropical Medicine, London, United Kingdom

**Keywords:** Sexual minority, Adolescence, Sexual behavior

## Abstract

**Purpose:**

North American research finds increased sexual risk-taking among teenagers with same-sex partners, but understanding of underlying processes is limited. The research carried out in the United Kingdom compares teenagers' early sexual experiences according to same- or opposite-sex partner, focusing on unwanted sex in addition to risk-taking, and exploring underlying psychosocial differences.

**Methods:**

Multivariate analyses combined self-reported data from two randomized control trials of school sex education programs (N = 10,250). Outcomes from sexually experienced teenagers (N = 3,766) were partner pressure to have first sex and subsequent regret, and sexual risk measures including pregnancy. Covariates included self-esteem, future expectations, substance use, and communication with mother.

**Results:**

By the time of follow-up (mean age, 16), same-sex genital contact (touching or oral or anal) was reported by 2.3% of teenagers, with the majority also reporting heterosexual intercourse. A total of 39% reported heterosexual intercourse and no same-sex genital contact. Boys were more likely to report partner pressure (Odds ratio [OR] = 2.56, 95% confidence intervals [CI] = 1.29–5.08) and regret (OR = 2.32; 95% CI = 1.39–3.86) in relation to first same-sex genital contact than first heterosexual intercourse, but girls showed no differences according to partner type. Teenagers with bisexual behavior reported greater pregnancy or partner pregnancy risk than teenagers with exclusively opposite-sex partners (girls, OR = 4.51, 95% CI = 2.35–8.64; boys, OR = 4.43, 95% CI = 2.41–8.14), partially reduced by attitudinal and behavioral differences.

**Conclusions:**

This UK study confirms greater reporting of sexual risk-taking among teenagers with same-sex partners, and suggests that boys in this group are vulnerable to unwanted sex. It suggests limitations to the interpretation of differences, in terms of psychosocial risk factors common to all adolescents.

See Editorial p. 5

There is mounting evidence from large-scale population studies of higher levels of sexual risk-taking among teenagers with same-sex partners, compared with teenagers with exclusively heterosexual relationships [1–6]. Currently, there has been limited exploration of underlying factors that might explain differences in early sexual risk-taking according to partner type. Apart from sexual risk, little is known about how experiences of early same-sex and opposite-sex sexual relationships compare. Moreover, evidence is confined to North American studies, although recent work suggests between-country variation in homophobia-related stresses and health consequences [7]. Interventions to address sexual health needs of young people with same-sex attractions would benefit from a clearer understanding of how these differ from those of the wider adolescent population.

There are two main aims of this study. The first is primarily descriptive. There are currently no large-scale quantitative data on young UK teenagers who have same-sex relationships, and prevalence information for teenagers aged under 16 depends on retrospective reports by an older age group [8,9]. This is the first UK study to compare the sexual experiences of teenagers according to whether they have opposite-sex or same-sex partners, combining two large representative school-based surveys. We examined both sexual risk and unwanted first experience, in terms of reported partner pressure to have sex and regret afterward. As associations between sexual orientation and risk may vary by gender, we look at effects for boys and girls separately [10–13].

The second aim of the study was to explore reasons for any differences in sexual risk-taking and unwanted sex according to partner type. Attempts to understand sexual risk-taking among adolescent sexual minority groups have adopted three main approaches. The first approach (minority stress theory) focuses on unique stressors experienced in developing a gay, lesbian, or bisexual identity [14,15]. This was the basis of a study finding associations between victimization at school and sexual risk [3]. A later study (exclusively of gay and bisexual youth) took account of a wider range of gay-related stressors and aspects of “coming-out,” finding associations between negative attitudes to homosexuality and sexual risk-taking [16]. Like many studies of sexual minority youth, it used a convenience, urban sample that may not be representative of the wider population. A more fundamental criticism is that research on sexual minority groups in isolation may mask risk factors that are common to all, regardless of sexual orientation [17,18].

Another approach focuses on sexual knowledge and skills deficits, but evidence is mixed and confined to nonrepresentative samples [19,20]. Such deficits could stem from limitations of school sex education programs [21,22]; less gay-sensitive sex education was associated with sexual risk in a representative U.S. school-based sample, but this did not take account of possible confounders in school and family environment [2]. The third approach is grounded in general theories of adolescent risk behavior suggesting multiple underlying psychosocial influences [23]. Here, evidence is limited to two studies of North American teenagers. One study (combining data from six school-based surveys) found that teenagers with same-sex attractions were disadvantaged with respect to school connectedness, liking for school, family connectedness, and religious identity, but did not attempt to link these to risk behaviors [24]. A separate study failed to find clear differences in academic orientation, friendship quality, and school climate according to sexual orientation, although teenagers with same-sex attraction were disadvantaged with respect to attitudes toward risk, psychosocial functioning, relationship with parents, and neighborhood quality [25]. A second phase of this research found that these factors acted as partial mediators for the effect of sexual orientation on an index of risk behaviors (including sexual risk), although a significant effect of minority orientation on increased risk remained [6].

Our study adopts a combination of the second and third approaches, asking whether any differences in sexual risk and unwanted first sex (FS) according to partner type are attributable to differences in sexual health knowledge and skills, as well as differences in psychosocial risk factors.

## Method

### Data collection

The analysis used data from the SHARE and RIPPLE studies, details of which have been published elsewhere [26,27]. A total of 25 schools participated in the SHARE randomized controlled trial of enhanced teacher-led sex education in Scotland. This trial was approved by Glasgow University's Ethical Committee for Non-Clinical Research Involving Human Subjects. A total of 27 schools participated in the RIPPLE randomized control trial of peer-led school sex education in England. This trial was approved by the Committee on the Ethics of Human Research at University College London. We combined data gathered from the two cohorts in both studies at baseline (SHARE 1996–1997, mean age: 14 years, 2 months; RIPPLE 1998–1999, mean age: 13 years, 8 months) and follow-up (SHARE 1998–1999, mean age: 16 years, 1 month; RIPPLE 2000–2001 mean age: 16 years, 0 months). SHARE baseline data were representative of the 1991 census of people living in Scotland in terms of parental social class and family composition. RIPPLE baseline data were representative of 1991 census English population data in terms of privately owned accommodation, and of 1998 General Certificate of Education qualifications (Examinations generally taken by secondary school pupils aged 14–16 years in England, Wales, and Northern Ireland).

Pupils completed questionnaires in their classrooms under examination conditions, administered by researchers only (SHARE) or teachers and researchers (RIPPLE). Early school leavers in the SHARE study completed postal questionnaires.

At follow-up, teenagers were asked whether they had experienced kissing with tongues and genital contact (two sets of questions, for opposite-sex and same-sex partners), and vaginal intercourse (with opposite-sex partner). Genital contact with an opposite-sex partner combined information from two questions on touching genitals and oral sex. Genital contact with a same-sex partner combined information from questions on touching genitals (RIPPLE and SHARE) and “had sex (any other activity involving genitals/private parts)” (in RIPPLE) or questions on oral sex and (for boys only) anal sex (in SHARE).

### Main outcomes

#### Unwanted FS

Information on partner pressure and regret was gathered in relation to first vaginal intercourse with an opposite-sex partner and first genital contact with a same-sex partner (both defined here as FS). For partner pressure, respondents were asked whether any pressure had been exerted, using a scale from “I put a lot of pressure on her or him” through “there was no pressure either way” to “she or he put a lot of pressure on me.” A binary variable grouped “no pressure either way” with respondent pressure, contrasting these responses with partner pressure. This exclusive focus on partner pressure, rather than any pressure (from respondent or partner) as a measure of unwanted sex from the respondent perspective comes from research on teenage heterosexual behavior indicating no differential effect of respondent pressure on regret or enjoyment of early sex [28]. Further analysis on teenagers reporting same-sex partners confirmed that regret did not vary according to whether respondent pressure was reported.

Regret was derived from a question about feelings after FS. A binary measure contrasted the responses “I wish I had waited longer” and “it shouldn't have happened at all” (taken to express regret) with “I wish I'd not waited so long” or “it was at about the right time.”

#### Sexual risk

There were five measures for all teenagers reporting vaginal intercourse with an opposite-sex partner: age at FS, condom use at first and most recent intercourse, number of partners in the past year, and pregnancy or (for boys) partner pregnancy. There were no measures of risk-taking with a same-sex partner in the combined data set.

### Key independent

The key independent was partner type. For models of unwanted sex, we compared teenagers reporting first same-sex genital contact with teenagers reporting heterosexual intercourse only. For models of sexual risk, we compared teenagers reporting bisexual behavior (heterosexual vaginal intercourse and same-sex genital contact) with teenagers reporting heterosexual intercourse only.

### Covariates

#### Sociodemographic factors

Baseline univariate comparisons indicated that the same-sex group contained higher proportions of teenagers (*p* < .05 for boys) from ethnic minority groups and families without both biological parents. There were no differences between partner-type groups according to a proxy measure for parental social class (social rented housing). Because ethnicity and family composition were associated with risk outcomes, we adjusted all multivariate analyses for these covariates.

#### Context of sexual behavior

First same-sex genital contact and first heterosexual intercourse are not equivalent events, and we adjusted for age at the time and having no expectation of sex to increase the validity of the comparison. A binary measure, “no expectation of sex,” was derived from agreement with either of the circumstances “It just happened on the spur of the moment” or “It was completely unexpected,” contrasted with agreement with any of “I expected it to happen soon, but was not sure when” or “I planned it to happen beforehand or “We planned it together beforehand.”

#### Attitudinal and behavioral confounders

Potential confounders of differences in sexual outcomes according to partner type comprised baseline attitudinal and behavioral measures. These comprised attitudes to school (scale using four items; Cronbach's alpha, .63); self-esteem (scale using three items; Cronbach's alpha, .66); substance use (scale using three items; Cronbach's alpha, .75); expectations of tertiary education and early parenthood, ease of communication with mother and father, and religiosity (five individual items coded 1–5); sexual health knowledge (scale using five true/false items); attitudes to condoms (scale using three items, Cronbach's alpha .70); and condom self-efficacy (scale using three items, Cronbach's alpha .69).

### Data analysis

From 12,500 teenagers who supplied information at follow-up, 10,250 were eligible for this analysis after excluding SHARE teenagers who were not asked about same-sex relationships (2,109 from nine schools in one education authority, plus a further 151 school leavers who completed a shorter postal questionnaire).

There were two stages to multivariate modeling. The first stage investigated the effect of partner type on sexual outcomes, adjusting for sociodemographic factors and study design. Covariates included at the first stage were age at follow-up, ethnicity, family composition, study (RIPPLE/SHARE), and trial arm (intervention or control). (Neither study had found differences between intervention and control arms in prevalence of heterosexual intercourse or use of contraception. The RIPPLE study found a borderline effect of lower unintended pregnancy among girls in the intervention arm reported at age 16 (2.3% vs. 3.3%, *p* = .07), although there was no corresponding between-arm difference in the SHARE study [26,27]). For models of unwanted FS, we also included age at FS and expectation of having FS as covariates (as noted previously). The second stage explored potential confounders of associations between partner type and sexual outcomes, and we added baseline attitudinal and behavioral covariates. Results are reported separately for boys and girls. All multivariate analyses allowed for clustering by school and were corrected for differential attrition from baseline to follow-up using a weighting system, created separately for each study using inverse values from logistic models of baseline predictors of response.

First, we performed complete case analyses using Stata version 10 (StataCorp LP, Texas, USA). In all models, missing information was greater in teenagers reporting same-sex partners than for those with exclusively heterosexual partners. To decrease bias and increase the power of the analyses, we used multiple chained equations (ICE program, version 1.7.0, Stata module by Patrick Royston, Medical Research Council Clinical Trials Unit, London, UK) to impute missing values [29]. This reduction in bias is expected when the missing items to be imputed are “missing at random,” meaning that their values are comparable to those observed for each variable given the observed values of other variables used in the imputation model. We imputed data on same-sex outcomes only for those who reported same-sex genital contact, and on opposite-sex outcomes only for those reporting heterosexual intercourse. Clustering of pupils by school was ignored in the imputation for simplicity. We generated 20 imputed data sets, and estimates were combined across these [30,31].

## Results

Sample composition is shown in Table 1. There were significant (*p* < .001) between-study differences in the proportion of minority ethnic groups and those in social rented housing.

Of the eligible sample (N = 10,250), 3,766 teenagers reported sexual behavior with either a same-sex or an opposite-sex partner or both, and are included in multivariate analyses. Almost four in 10 teenagers (39.3%, N = 3,565 reported heterosexual intercourse without any report of same-sex behavior, and 2.3% (N = 201) reported same-sex genital contact (Table 1). Most teenagers reporting same-sex genital contact had also experienced heterosexual intercourse (last row of table, for combined sexes the bisexual group, 1.6% of the sample, N = 137, comprised 72% of those with same-sex partners, allowing for weighting). A minority of participants reporting a same-sex partner (nine of 201) did not answer questions concerning opposite-sex partners and are treated in these analyses as having same sex partners only. Similarly, 330 participants reported an opposite-sex partner but did not answer questions concerning same-sex partners and are treated as having opposite-sex partners only.

Girls were more likely than boys to report same-sex kissing with tongues and heterosexual intercourse (both *p* < .001), but there were no other gender differences in reporting of sexual behavior. Although a slightly higher percentage of SHARE teenagers reported heterosexual intercourse than in the RIPPLE study (*p* < .01), there were no other significant (*p* < .05) between-study differences in rates of other sexual behaviors with same- or opposite-sex partners.

Among boys, the prevalence of unwanted FS was higher for first homosexual genital contact than for first heterosexual intercourse in the exclusively heterosexual group (Table 2). Among girls, there were no differences in rates of unwanted sex according to partner type. In boys and girls, the prevalence of sexual risk-taking was higher for those with partners of both sexes, as compared with teenagers with exclusively opposite-sex partners. Similar effects of partner type were apparent in both the RIPPLE and SHARE studies when examined separately (not shown).

We now consider attitudinal and behavioral factors reported at baseline (age 13 or 14 years) that may confound differences in sexual outcomes. Univariate analyses revealed some significant (*p* < .05) or borderline significant (*p* < .08) differences in attitudinal and behavioral measures according to partner type (Table 3). Teenagers with same-sex partners were more religious and more knowledgeable about sexual health, and (boys) were more likely to expect tertiary education than the exclusively heterosexual group. Boys with same-sex partners had lower self-esteem, and girls reported poorer communication with their mother. Most of these differences were also seen when comparing teenagers reporting bisexual behavior with exclusively heterosexual counterparts. Overall, several factors in the bisexual group were protective against sexual risk-taking (greater knowledge, religiosity, and expectations of tertiary education). However, girls with bisexual behavior reported factors associated with greater sexual risk (poor communication with mother, substance use, and expectation of early parenthood).

Results are provided for stage one multivariate analysis using both complete case information and the imputed data set. Coefficients/odds ratios are similar, although for pressure and regret outcomes the imputed data set shows a greater risk associated with same-sex partner for boys. This is consistent with a reduction in bias because of lower disclosure of negative experiences by teenagers with same-sex partners. In this study, we describe results using the imputed data set.

### Unwanted FS

Partner pressure and regret were compared for first same-sex genital contact and opposite-sex intercourse (among teenagers *not* reporting same-sex genital contact). The latter group were older than the same-sex group (mean ages respectively, 14.4 years, SD: 1.15 and 13.4 years, SD: 2.9, *p* < .001), and were more likely to have expected sex (55% vs. 25%, *p* < .001). Age and expectation of sex were strongly associated with the two outcomes, and were included as covariates at stage 1 (Table 4). There was a strong gender difference in the effect of partner type. Boys with a same-sex partner were more likely to report partner pressure and regret, although there was no effect of partner type among girls. The only potential confounder for the effect of partner type on unwanted sex among boys arising from univariate analyses in Table 3 was self-esteem. However, there was only a small effect of adjusting for self-esteem on odds associated with same-sex partner in stage 2, Table 4.

Dividing the same-sex partner group and comparing again with boys reporting opposite-sex partners only (not in Table 4), the effects were similar for boys reporting same-sex genital contact only (pressure: OR = 2.11, 95% CI = .75–5.91; regret: OR = 3.73, 95% CI = 1.51–9.25) and boys who reported bisexual behavior (pressure: OR = 2.80, 95% CI = 1.23–6.35; regret: OR = 1.79, 95% CI = 1.00–3.22).

The RIPPLE data set contained a wider range of contextual measures, and indicated that same-sex encounters were more likely to involve alcohol or drugs and no prior partner relationship, although no more likely to involve an older partner. Further exploration (not shown) confirmed boys' greater likelihood of negative feelings after first same-sex genital contact, taking account of additional contextual information.

### Sexual risk

Sexual risk was compared for teenagers reporting bisexual behavior and those reporting heterosexual intercourse only (Table 5, stage 1). Bisexual behavior was significantly associated with greater risk (boys: three measures, girls: four measures). Baseline differences in early parenthood, substance use, and poor communication with mother appeared to be potential confounders of these effects among girls (Table 3). For girls, effects of partner type were reduced but remained significant after adding these covariates in stage 2, Table 5. For boys, there was less effect of adding baseline covariates.

Further adjusting the pregnancy models for characteristics of sexual behavior (age and partner pressure at first heterosexual intercourse, number of partners, not shown in Table 5) attenuated the risk associated with bisexual behavior to nonsignificance among girls (OR = 1.85, 95% CI = .98–3.51), but not among boys (OR = 3.53, 95% CI = 1.86–6.67).

## Discussion

This UK study found that bisexual behavior in teenage boys and girls was associated with greater sexual risk-taking than exclusively heterosexual behavior, including a more than three-fold increase in pregnancy/partner pregnancy odds. This risk-taking accords with previous studies of teenagers [4,5,32] and older populations [9]. We also found that boys with a same-sex partner were more vulnerable to unwanted FS, reporting greater partner pressure and regret than their exclusively heterosexual counterparts. Boys' reported partner pressure appears in line with low relationship control reported by sexual minority boys in a U.S. study [33]. Sexual minority boys were more likely than girls to report sexual coercion in seven North American population-based surveys [34], but the extent of physical coercion, victimization, or sexual abuse in our measure is unknown.

We explored potential confounders of differences in sexual outcome according to partner type. Low statistical power prevented us from excluding cases where baseline covariates postdated FS, so there may have been an element of reverse causation. With regard to risk-taking, there was little evidence of condom attitude or skills deficits, and sexual health knowledge was higher among the bisexual group; this contrasts with more mixed findings elsewhere [19,20]. There was some evidence for more general psychosocial confounders of risk-taking, especially among girls (difficult communication with mother, future expectations of early parenthood, and substance use). However, in both sexes the effect of partner type on sexual risk-taking remained after taking account of psychosocial confounders. This echoes the results of a North American study [6] that found significant effects of sexual orientation group on adolescent risk-taking after taking account of psychosocial mediators.

Our finding of greater unwanted sex among boys with same-sex partners held after adjusting for baseline self-esteem and important differences in the circumstances of same-sex and opposite-sex encounters. Our finding mirrors gender differences in approval of same-sex relationships, reported elsewhere among teenagers in the United Kingdom [35]. Boys' greater disapproval of gay male relationships suggests an explanation for regret.

The study suffers from several limitations, notably its use of self-reported measures of sensitive behavior [36]. In general, inclusion of questions regarding same-sex behavior appeared acceptable to both schools and young people, although one education authority in the SHARE study refused to allow these questions. Comments at the end of the questionnaire suggest that some teenagers welcomed the opportunity to report on such behavior. However, although young people were asked to complete the questionnaire without talking to friends, researchers frequently observed young people, particularly boys, making homophobic comments. Rates of missing responses for detailed questions about same-sex experiences were greater than for equivalent opposite-sex experiences, suggesting a reluctance to divulge more sensitive information despite reassurances of confidentiality. Imputation of missing items using predictors (including partner type) helped to overcome risk of bias and loss of power inherent in complete case analyses. The risk of bias in both studies due to differential attrition from baseline to follow-up was addressed through the use of weights, which make it more likely that the results generalize to a wider population of teenagers. Rates of same-sex sexual behaviors found in 15–16 year olds were comparable with retrospective reports in national surveys of older UK respondents; these also confirm our finding that most with same-sex partners also experience heterosexual intercourse [8,9].

Our study is confined to the early sexual experiences of a young age group. More research is needed to establish whether our findings extend to subsequent sexual experiences and to those initiating sexual relationships at an older age. Further research should include measures of sexual risk in same-sex encounters. We use a behavioral classification of sexual orientation rather than a measure of sexual attraction or identity: discordance between such measures during adolescence is well known, and future research should use multiple orientation measures [5,37]. A further limitation is the age of our data set, since over the last decade the UK has seen greater social tolerance and legitimization of same-sex relationships [38]. Nevertheless, recent evidence suggests that homophobic bullying and victimization among school-age teenagers are still commonplace in the UK and U.S. [39,40].

This article extends the evidence base on early same-sex behavior to a UK setting, and describes unwanted sex in addition to risk-taking. The results confirm the unique vulnerability of teenagers with same-sex partners, and suggest limitations to the interpretation of differences using psychosocial risk factors common to all adolescents. Greater understanding in future research might come from the application of measures designed to capture gay-related stressors, such as bullying and fear of stigmatization.

## Figures and Tables

**Table 1:** Sample characteristics: socio-demographic information and sexual behavior according to partner type

	Both sexes			Boys			Girls		
	Combined data sets (N = 10,250) %	RIPPLE (N = 6,656) %	SHARE (N = 3,594) %	Combined data sets (N = 5,077) %	RIPPLE (N = 3,426) %	SHARE (N = 1,651) %	Combined data sets (N = 5,173) %	RIPPLE (N = 3,230) %	SHARE (N = 1,943) %
Sociodemographic information									
Family composition									
Do not live with both biological parents	29.1	28.0	31.1	27.4	26.9	28.3	30.8	29.1	33.6
Ethnic group									
Non-white	11.2	15.7	3.1	12.9	17.9	3.1	9.4	13.3	3.1
Housing									
Social rented	29.5	27.7	32.5	27.4	27.0	31.0	30.5	28.5	33.8
Sexual behaviors reported at follow-up, aged 15/16 years									
Kissing with tongues									
Opposite-sex partner only	90.6	93.8	85.6	90.9	94.5	84.9	90.4	93.2	86.2
Any same-sex partner	3.5	4.0	2.8	2.3	2.4	2.2	4.6	5.6	3.2
Same-sex partner only	.2	.3	.2	.3	.4	.2	.1	.2	.1
Partners of both sexes	3.3	3.7	2.6	2.0	2.0	2.0	4.5	5.4	3.1
Genital contact (petting)									
Opposite-sex partner only	68.7	67.9	70.1	67.7	67.8	67.7	69.6	66.4	70.5
Any same-sex partner	2.3	2.1	2.4	2.3	2.0	2.7	2.3	2.3	2.2
Same-sex partner only	.3	.2	.3	.4	.4	.4	.2	.1	.2
Partners of both sexes	2.0	1.9	2.1	1.9	1.6	2.3	2.1	2.2	2.0
Oral sex^a^									
Opposite-sex partner only			38.4			34.5			41.9
Any same-sex partner			1.4			1.9			1.0
Same-sex partner only			.4			.6			.3
Partners of both sexes			1.0			1.3			.7
Vaginal/anal intercourse^a^									
Opposite-sex partner only						38.1			
Any same-sex partner						1.1			
Same-sex partner only						.1			
Partners of both sexes						1.0			
“First sex”^b^									
Opposite-sex partner only (vaginal intercourse)	39.3	43.6	37.0	35.1	33.3	38.8	43.4	40.8	47.9
Any same-sex partner (genital contact)	2.3	2.3	2.4	2.2	2.0	2.7	2.3	2.4	2.3
Same-sex partner only (no vaginal intercourse with opposite-sex partner)	.6	.7	.6	.7	.8	.7	.5	.5	.6
Partners of both sexes (genital contact with same-sex partner, vaginal intercourse with opposite-sex partner)	1.6	1.6	1.8	1.5	1.2	2.0	1.8	1.9	1.7

a Information on oral sex and (boys) anal intercourse with a same-sex partner was not collected in the RIPPLE study.

b Defined as genital contact for same-sex partner, and as vaginal intercourse for opposite-sex partner. The division between those with “same sex partner only” and those with “partners of both sexes” differs from that shown for petting, since not all teenagers reporting petting with partners of both sexes also experienced heterosexual intercourse.

**Table 2:** Prevalence of unwanted first sex and sexual risk according to partner type, by gender: univariate comparisons

	Boys			1 vs. 3	2 vs. 3	Girls			1 vs. 3	2 vs. 3
	(1) Any genital contact with same-sex partner	(2) Any same-sex genital contact, and heterosexual intercourse	(3) Heterosexual intercourse only			(1) Any genital contact with same-sex partner	(2) Any same-sex genital contact, and heterosexual intercourse	(3) Heterosexual intercourse only		
Base N	101	63	1,557			100	74	2,008		
Sexual outcomes	% of sexual group with outcome at first same-sex genital contact	% of sexual group with outcome at first same-sex genital contact	% of sexual group with outcome at first heterosexual intercourse	*p*	*p*	% of sexual group with outcome at first same-sex genital contact	% of sexual group with outcome at first same-sex genital contact	% of sexual group with outcome at first heterosexual intercourse	*p*	*p*
Unwanted first sex										
Partner pressure FS	26	29	9	.001	.001	25	25	19	.221	.260
Regretted FS	44	41	23	.001	.003	50	48	42	.186	.362
		% of sexual group with outcomes related to heterosexual intercourse	% of sexual group with outcomes related to heterosexual intercourse				% of sexual group with outcomes related to heterosexual intercourse	% of sexual group with outcomes related to heterosexual intercourse		
Sexual risk										
Age under 13 years at first heterosexual intercourse		13	6		.073		8	3		.028
No condom FS		41	27		.012		41	31		.044
No condom LS		33	30		.587		62	43		.002
Three or more opposite-sex partners in last year		58	23		.001		38	22		.002
Pregnancy/partner pregnancy		24	7		.001		34	9		.001

N values show raw data, percentages show weighted values. Probabilities show results of Chi-square tests.

**Table 3:** Attitudinal and behavioral differences according to partner type, by gender: univariate analyses

	Boys			1 vs. 3	2 vs. 3	Girls			1 vs. 3	2 vs. 3
	(1) Any genital contact with same-sex partner	(2) Any same-sex genital contact, and heterosexual intercourse	(3) Heterosexual intercourse only			(1) Any genital contact with same-sex partner	(2) Any same-sex genital contact, and heterosexual intercourse	(3) Heterosexual intercourse only		
*Base N*	101	63	1,557			100	74	2,008		
Measures collected at baseline, aged 13 or 14 years										
Direction of coding	Mean (SD)	Mean (SD)	Mean (SD)	*p*	*p*	Mean (SD)	Mean (SD)	Mean (SD)	*p*	*p*
Attitudes to school										
High = more positive attitudes	3.40 (.80)	3.28 (.83)	3.26 (.78)	.080	.845	3.34 (.73)	3.22 (.72)	3.32 (.71)	.758	.208
Expectation of tertiary education										
High = greater expectation	3.86 (1.05)	3.79 (1.18)	3.46 (1.11)	.001	.016	3.70 (1.12)	3.51 (1.13)	3.66 (.99)	.664	.176
Expectation of early parenthood										
High = lower expectation	3.42 (1.25)	3.34 (1.31)	3.29 (1.16)	.278	.700	3.43 (1.16)	3.29 (1.17)	3.52 (1.16)	.449	.073
Ease of communication with mother										
High = greater comfort/ease	2.84 (1.33)	2.92 (1.36)	2.82 (1.37)	.911	.574	3.08 (1.43)	3.06 (1.47)	3.39 (1.26)	.028	.047
Ease of communication with father										
High = greater comfort/ease	2.74 (1.41)	2.95 (1.43)	2.90 (1.43)	.309	.783	2.05 (1.30)	2.03 (1.32)	1.99 (1.16)	.629	.795
Self-esteem										
High = greater self-esteem	3.47 (.73)	3.51 (.75)	3.64 (.61)	.024	.167	3.14 (.82)	3.13 (.86)	3.27 (.70)	.106	.127
Religiosity										
High = greater religiosity	2.19 (1.31)	2.23 (1.39)	1.91 (1.11)	.038	.065	2.44 (1.19)	2.36 (1.16)	1.97 (1.05)	.001	.004
Substance use										
High = less substance use	3.17 (.79)	3.02 (.82)	3.13 (.76)	.278	.275	2.98 (.81)	2.80 (.80)	2.96 (.79)	.761	.064
Knowledge of sexual health										
High = greater knowledge	3.34 (1.59)	3.56 (1.54)	2.90 (1.61)	.006	.001	3.58 (1.46)	3.59 (1.55)	3.31 (1.54)	.071	.100
Attitudes to condoms										
High = more positive attitudes	3.57 (.87)	3.64 (.90)	3.48 (.94)	.377	.204	3.88 (.95)	3.89 (.97)	3.81 (.80)	.391	.457
Condom self-efficacy										
High = greater self-efficacy	4.03 (.75)	4.13 (.65)	4.07 (.71)	.625	.470	3.80 (.89)	3.93 (.80)	3.80 (.70)	.962	.165

N values show raw data, percentages show weighted values. Probabilities show results of *t*-tests.

**Table 4:** Multivariate analysis of partner pressure and regret according to partner type, comparing first genital contact with same-sex partner with first heterosexual vaginal intercourse

Partner type	Partner pressure						Regret					
	Complete case (N = 1,010)		Imputed data set (N = 1,658)				Complete case (N = 1,018)		Imputed data set (N = 1,658)			
	Stage 1^a^		Stage 1^a^		Stage 2^b^		Stage 1^a^		Stage 1^a^		Stage 2^b^	
	OR (95% CI)	*p*	OR (95% CI)	*p*	OR (95% CI)	*p*	OR (95% CI)	*p*	OR (95% CI)	*p*	OR (95% CI)	*p*
Boys												
Opposite-sex partner only (vaginal intercourse)	1.00		1.00		1.00		1.00		1.00		1.00	
Same-sex partner (genital contact)	2.21 (.87–5.63)	.097	2.56 (1.29–5.08)	.008	2.52 (1.26–5.04)	.009	1.97 (1.06–3.68)	.033	2.32 (1.39–3.86)	.001	2.28 (1.37–3.79)	.002

	Complete case (N = 1,548)		Imputed data set (N = 2,108)				Complete case (N = 1,539)		Imputed data set (N = 2,108)			
	Stage 1^a^		Stage 1^a^		Stage 2^b^		Stage 1^a^		Stage 1^a^		Stage 2^b^	
	OR (95% CI)	*p*	OR (95% CI)	*p*	OR (95% CI)	*p*	OR (95% CI)	*p*	OR (95% CI)	*p*	OR (95% CI)	*p*
Girls												
Opposite-sex partner only (vaginal intercourse)	1.00		1.00		1.00		1.00		1.00		1.00	
Same-sex partner (genital contact)	.58 (.20–1.67)	.317	.69 (.28–1.68)	.412	.68 (.28–1.66)	.399	.48 (.21–1.09)	.080	.81 (.36–1.79)	.592	.80 (.36–1.77)	.580

a Adjusted for study, intervention/control group, sociodemograhics, age in months at follow-up, age at first sex and expectation of first sex.

b Further adjusted for baseline self esteem.

**Table 5:** Multivariate analysis of sexual risk according to partner type, comparing teenagers with both same- and opposite-sex partners and teenagers with opposite-sex partners only

Boys		Effect for bisexual behavior						
		Complete case analysis			Analysis using imputed data set (N = 1,620)			
Sexual risk outcomes reported at follow-up		N	Stage 1^a^	*p*	Stage 1^a^	*p*	Stage 2^b^	*p*
Age at first heterosexual intercourse	Coefficient (95% CI)	1,078	−.25 (−.68 to .18)	.242	−.33 (−.74 to .07)	.102	−.33 (−.74 to .07)	.103
No condom at first heterosexual intercourse	OR (95% CI)	1,307	1.96 (1.12–3.44)	.018	1.96 (1.13–3.40)	.017	2.02 (1.13–3.61)	.017
No condom at most recent heterosexual intercourse	OR (95% CI)	944	1.11 (.61–2.02)	.724	1.11 (.60–2.05)	.747	1.10 (.57–2.12)	.770
Number of partners in last 12 months	Coefficient (95% CI)	801	.51 (.02–1.00)	.041	.59 (.05–1.13)	.034	.59 (.05–1.13)	.034
Pregnancy/partner pregnancy	OR (95% CI)	1,237	4.21 (2.25–7.86)	.000	4.43 (2.41–8.14)	.000	3.09 (1.67–5.73)	.000

Girls								
		Complete case analysis		Analysis using imputed data set (N = 2,082)				
Sexual risk outcomes reported at follow-up		N	Stage 1^a^	*p*	Stage 1^a^	*p*	Stage 2^b^	*p*
Age at first heterosexual intercourse	Coefficient (95% CI)	1,658	−.57 (−.90 to −.24)	.001	−.52 (−.81 to −.22)	.001	−.41 (−.66 to −.15)	.003
No condom at first heterosexual intercourse	OR (95% CI)	1,774	1.56 (.97–2.51)	.069	1.58 (1.00–2.51)	.051	1.38 (.87–2.20)	.169
No condom at most recent heterosexual intercourse	OR (95% CI)	1,472	2.02 (1.07–3.83)	.031	2.12 (1.20–3.73)	.010	1.90 (1.06–3.39)	.031
Number of partners in last 12 months	Coefficient (95% CI)	1,293	.98 (.11–1.86)	.029	.92 (.21–1.63)	.013	.85 (.14–1.56)	.021
Pregnancy/partner pregnancy	OR (95% CI)	1,810	3.13 (1.72–5.72)	.000	4.51 (2.35–8.64)	.000	2.66 (1.47–4.82)	.001

a Adjusted for study, intervention/control group, gender, sociodemographics and age in months at follow-up.

b Further adjusted for parenthood expectations, ease of communication with mother and substance use.
